# What does the structure-function relationship of the HIV-1 Tat protein teach us about developing an AIDS vaccine?

**DOI:** 10.1186/1742-4690-6-50

**Published:** 2009-05-25

**Authors:** Grant R Campbell, Erwann P Loret

**Affiliations:** 1 Department of Pediatrics, Division of Infectious Diseases, University of California San Diego, 9500 Gilman Drive, La Jolla, California 92093-0672, USA; 2 Unité Mixte de Recherche Université de la Méditérranée/Institut National de la Santé et de la Recherche Médicale U911, Faculté de Pharmacie, 27 Bd Jean Moulin, 13385 Marseille, France

## Abstract

The human immunodeficiency virus type 1 (HIV-1) trans-activator of transcription protein Tat is an important factor in viral pathogenesis. In addition to its function as the key trans-activator of viral transcription, Tat is also secreted by the infected cell and taken up by neighboring cells where it has an effect both on infected and uninfected cells. In this review we will focus on the relationship between the structure of the Tat protein and its function as a secreted factor. To this end we will summarize some of the exogenous functions of Tat that have been implicated in HIV-1 pathogenesis and the impact of structural variations and viral subtype variants of Tat on those functions. Finally, since in some patients the presence of Tat-specific antibodies or CTL frequencies are associated with slow or non-progression to AIDS, we will also discuss the role of Tat as a potential vaccine candidate, the advances made in this field, and the importance of using a Tat protein capable of eliciting a protective or therapeutic immune response to viral challenge.

## Review

### Introduction

Human immunodeficiency virus type 1 (HIV-1) exhibits high genetic variability, with strains divided into three main groups: major (M), which are the cause of most HIV-1 infections worldwide, outlier (O) and new (N) that are non M and non O [1]. Within group M, nine subtypes are recognized, designated by the letters A-D, F-H, J and K. In addition, circulating recombinant forms (CRF) have also been identified [1]. Globally, over 50% of all infections are caused by subtype C which is found mainly in sub-Saharan Africa, India and South America, whereas subtype B, the most studied clade, represents 10% of all infections, and is dominant in both Europe and America. Subtypes A and D are found in sub-Saharan Africa and account for 12% and 3% of infections respectively, while CRF_01_AE is found mainly in south east Asia and represents 5% of all infections worldwide [1]. Recent research has shown that the different subtypes and CRF of HIV-1 have biological differences with respect to transmission [2], replication [3] and disease progression [4,5]. Moreover, the HIV-1 proteins gp120 [6], Nef [7], Vif, Vpr, Vpu [8,9] and Tat [10-19] show clade and isotype-specific properties at both the molecular and biological levels. Therefore, a generalization of our understanding of HIV-1 subtype B transmission, pathogenesis and tissue involvement across all subtypes is questionable.

The HIV-1 *trans*-activator of transcription (Tat) is an 86–101 residue regulatory protein (9–11 kDa) that is essential for the productive and processive transcription from the HIV-1 long terminal repeat (LTR) promoter [20-22]. Tat binds to a short nascent stem-bulge loop leader RNA, termed the *trans*-activation responsive region, or TAR [23,24], that is present at the 5' extremity of all viral transcripts via its basic region and recruits the complex of cyclin T1 and cyclin-dependent kinase 9 (CDK9) forming the positive transcription elongation factor B complex. CDK9 hyperphosphorylates the carboxy terminus domain of RNA polymerase II, leading to the enhanced elongation of transcription from the viral promoter. For Tat's transcriptional activity, it has recently been reported that Tat is regulated by lysine methylation [25], and that it interacts with a histone chaperone nucleosome assembly protein [26].

In addition to its primary role as a transcriptional activator of viral gene expression, Tat is actively released from unruptured, HIV-1-infected cells and is detectable in *ex vivo *culture supernatants and in the serum of HIV-1 infected individuals at concentrations up to 40 ng/mL [27,28]. This exogenous Tat is able to enter both uninfected and latently infected cells, inducing apoptosis in the former and activating the transcription of the viral genome in the latter. The precise mechanism by which Tat enters cells is under investigation and will not be discussed here. However, no specific receptor has been implicated in the uptake of Tat and conflicting results have been obtained for the involvement of macropinocytosis [29], clathrin-mediated endocytosis [30] and caveolae/lipid-raft-mediated endocytosis [31]. Thus, Tat fulfills a role in HIV-1 pathogenesis not only as an essential protein for HIV-1 replication, but also as an extra-cellular toxin [32]. Therefore, it is relevant to develop a vaccine targeting Tat [33]. However, antibodies against Tat are found in almost 50% of seropositive patients but are unable to recognize Tat variants from all HIV-1 subtypes [17]. Moreover, these antibodies fail to slow disease progression to AIDS [34].

Understanding the structure-function relationship in respect to the exogenous roles of Tat may have important clinical implications, both for the development of new vaccines against AIDS targeting Tat. Here, we present the latest advances in elucidating the structure of Tat. We will also summarize some of the roles exogenous Tat has been shown to fulfill, and the impact that structural variations of Tat may have on these functions. Finally, we will also discuss the role of Tat as a potential vaccine candidate.

### Structures of Tat variants

HIV-1 Tat is a small nuclear protein that exists predominantly in two different lengths – 86–87 residues or 99–101 residues – and is encoded by two exons [20]. The long 99–101 residue forms are predominant in clinical isolates from all HIV-1 subtypes excepted subtype D, which has a non-synonymous single nucleotide polymorphism, creating a stop codon in the second exon encoding sequence. However, some subtype B isolates have been found that have this truncated form, and is the form of Tat most used in research [15,20]. Tat is divided into six regions [35] with the one termed the basic region being involved in most of Tat's functions [20]. Nuclear magnetic resonance spectroscopy (NMR) studies of biologically active Tat variants revealed that the basic region and the other functional regions are well exposed to solvent and surround a core composed of part of the N-terminus, where the well conserved Trp^11 ^is found [36-38]. This folding is similar between different Tat variants in aqueous solution but can change dramatically when exposed to hydrophobic solvents [10]. Tat is a flexible protein, and structural changes are probably necessary for it to bind to its pharmacological targets [39].

#### Primary structure

**Figure 1:**
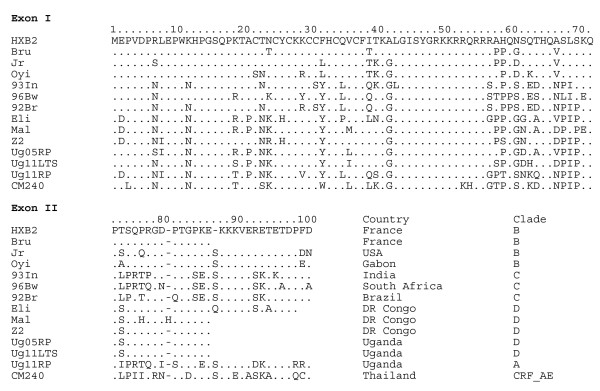
**Tat sequences representative of the five main HIV-1 subtypes**. The sequence length of Tat is variable and ranges from 86 to 101 residues as a function of the second exon. A viable strain having only the first exon of Tat (72 residues) has never been observed *in vivo*. Subtype variability follows the geographical diversity of HIV-1 with subtype B Tat sequences being the most divergent compared to subtypes A, C, D and CRF_AE. These Tat variants have been synthesized using solid phase synthesis and have been shown to be able to cross membranes and *trans*-activate the HIV-1 LTR except for Tat Oyi [10,14-16,41,52].

Tat was first described as a trans-activator of HIV-1 genes [40]. Although *trans*-activation can be observed *in vitro *with the first exon (residues 1–72), the second exon that codes for 14 to 34 amino acids at the C-terminal extremity is necessary to observe *trans*-activation *in vivo *[20]. Figure 1 shows a selection of Tat sequences obtained using solid phase synthesis [10] that all have *trans*-activational transcription activity (excepted Tat Oyi). This data show that Tat can tolerate up to 40% sequence variation without loss of activity [41].

Tat is divided into six different functional regions [35]. Region I (residues 1–21) is a proline-rich region and has a conserved Trp^11^. Region II (residues 22–37) has seven well conserved cysteines at positions 22, 25, 27, 30, 31, 34 and 37 except for subtype C which has a C31S mutation. These cysteines appear to be free and no other cysteines are found in the sequence except in CRF_01_AE (Figure 1) and CRF_01_AG [42]. It was proposed that a functional Tat could have cysteines bound to zinc [40]. The functional test was the *in vitro *modulation of microtubule assembly but a same effect is obtained with a Tat peptide (residues 38–72) that does not contain the cysteine rich region [18]. The *trans *activation assay *in vivo *with different synthetic Tat variants does not require zinc binding [10]. Region III (residues 38–48) has a conserved Phe^38 ^and the conserved sequence ^43^LGISYG. Region IV (residues 49–59) is rich in basic residues and has the rather well conserved sequence ^49^RKKRRQRRRPP. Region V (residues 60–72) is the glutamine-rich region and has the highest rate of sequence variation. Region VI constitutes the C-terminus of Tat, is encoded by the second exon, and contains a conserved RGD motif in subtypes B and D [20].

#### Secondary Structure

Circular dichroism reveals that the main secondary structure in aqueous solution is the *β*-turn with an average of 30% among Tat variants and almost no *α*-helix [10]. However, the secondary structures of Tat are dependent upon its environment and change dramatically with an *α*-helix becoming the main secondary structure in hydrophobic solvents [10]. These changes reveal that Tat is highly flexible, and this is almost certainly related to the capacity of Tat to cross cell membranes.

**Figure 2:**
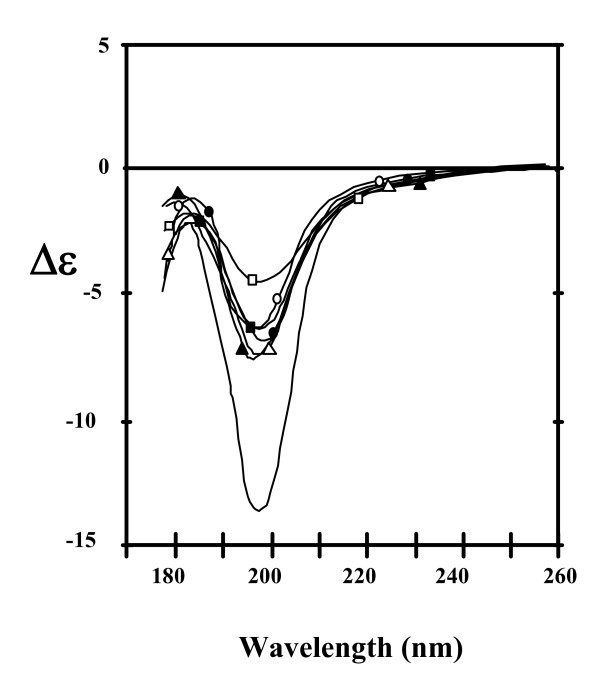
**Circular dichroism (CD) spectra of Tat variants in aqueous solution**. Tat Z2 (white triangle), Tat Oyi (black triangle), Tat Bru (white circle), Tat Bru cmC (no mark), Tat Jr (black circle), Tat Mal (white square) and Tat Eli (black square) were measured from 260 to 178 nm with a 50 μM path length in 20 mM phosphate buffer, pH 4.5. It is not possible to gather CD spectra into two categories composed of short Tat (white mark) or long Tat (black mark). The intense magnitude of the 200 nm band observed with Tat Bru cmC shows that chemical modifications of cysteines modify the folding of Tat.

Peptides corresponding to the different Tat regions show the same capacity of change in the secondary structures with respect to its environment as the native protein [43]. However, regions I and VI are less flexible, probably due to their high proline content (Figure 1). Interestingly, region III seems to be the only one able to adopt a *β*-turn structure independently from the other regions [43]. Chemical modification of the seven cysteines dramatically changes the CD spectrum of Tat Bru (Figure 2) revealing significant structural changes [10].

#### Tertiary structure

**Figure 3:**
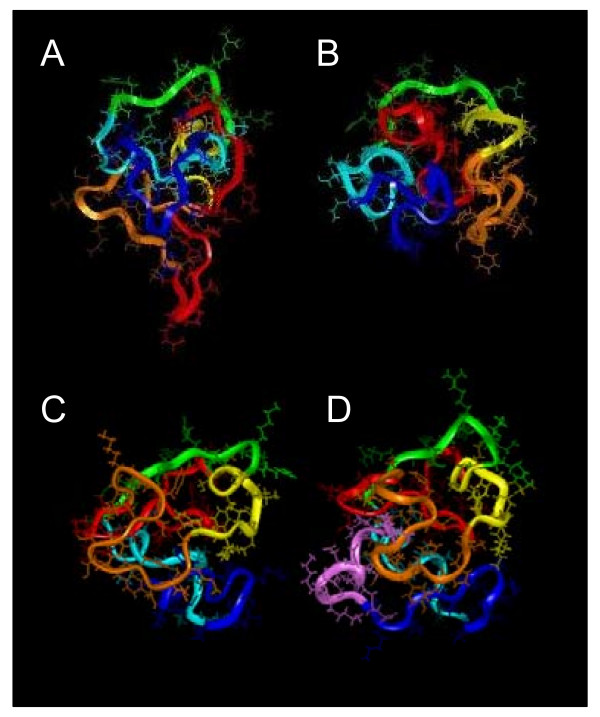
**NMR studies of Tat proteins**. Tat Z2 (A), Tat Bru (B), Tat Mal (C), and Tat Eli (D) 3D structures obtained from NMR constraints [36-38,44]. Region I is depicted in red, region II (cysteine-rich region) in orange, region III in yellow, region IV (basic region) in green, region V in light blue, region VI (residues 73–86/87) in blue and for Tat Eli the extra C-terminal residues are in pink. The Tat Z2 variant used had chemically modified cysteines which affected biological activity and 3D structure. The three Tat variants with biological activity (B, C and D) displayed a similar folding characterized by a core region composed of part of region I with the highly conserved Trp^11 ^while the functional region II, IV and V are well exposed to the solvent. The extra residues in the C-terminus of Tat Eli are exposed to the solvent and protrude from a groove between the basic region and the cysteine-rich region.

No X-ray crystallography structural studies of a full length Tat have been performed, but four NMR studies of Tat variants with two exons have been reported (Figure 3). The first NMR structural study was performed under reducing conditions using an 86-residue Tat Z2 variant in the presence of dithiothreitol (DTT) [44]. The oxidation state of the cysteine residues is important when considering Tat's *trans*-activational function as Tat becomes inactive when incubated with strong reducing agents such as DTT or 2-mercaptoethanol [45]. Furthermore, chemical modification of cysteines changes dramatically the CD spectrum of Tat [10]. Only 25 long distance NMR constraints, mainly located in regions III and V were obtained in this study [44]. Two later studies of the 86-residue Tat Bru [36] and the 87-residue Tat Mal [37] were performed in the absence of reducing agents and over 270 long-range NMR constraints were found in each. Both Tat proteins displayed different folding to that of Bayer et al. [44] but similar to each other. Tat Mal has a sequence similar to Tat Z2 (Figure 1), and the CD spectrum of Tat Z2 in the absence of reducing agents is similar to that of both Tat Mal and Tat Bru; both of which have been shown to be biologically active in the absence of reducing agents. Therefore, it is probable that the different folding observed in the NMR study of Tat Z2 (Figure 3A) is due to a structural change induced by the reducing conditions. An NMR study of a reduced peptide corresponding to the first exon of Tat (residues 1–72) combined with a His_6 _segment and T7 epitope that added 20 residues to the N-terminus resulting in a 92-residue peptide has also been performed recently [46]. In this case, the authors were unable to identify NMR constraints and stated that Tat was a naturally unfolded protein. It is surprising to deduce this statement for all Tat variants from the study of a 72 residue reduced Tat-His_6_T7 peptide as no viable HIV-1 strain consisting of only the first exon of Tat has ever been observed *in vivo*. Furthermore, the sequence used for this study does not correspond to a viable HIV-1 strain, as the peptide contained a supplemental 20 residues at the N-terminus that are unrelated to Tat. The *trans*-activational activity of this peptide was not tested or its ability to induce TNF production from monocytes; so it is not possible to determine if this study was biologically relevant. Moreover, a conserved Tat folding is also confirmed by numerous vaccine studies that raised antibodies against Tat conformational epitopes in HIV-1-infected individuals and SHIV-1-infected macaques [17,47-50]. Taken together, these findings indicate that Tat with its two exons should exist in a stable conformation *in vivo*. Furthermore, the second exon of Tat was is essential to get a biologically functional Tat in a number of different assays [41,51-53]. Therefore, the collective studies indicate that the second exon of Tat is important to the stability of the structure. The last NMR study of Tat to be reported was the first report of a NMR structure for a full-length Tat and was performed using the 99-residue Tat Eli variant [38]. Figure 3D shows that Tat Eli has a core region made up of a part of the N-terminus with the highly conserved Trp^11 ^and a folding similar to Tat Bru and Tat Mal with the extra residues at the Tat Eli C-terminus protruding from a groove between the basic region and the cysteine-rich region that is well exposed to solvent [38].

The main secondary structure building block in Tat variants is the *β*-turn [36-38]. The core of Tat is composed primarily of aromatic residues organized in a hydrophobic cluster involving the highly conserved Trp^11 ^and Phe^38^, with a part of region I adopting an extended structure that crosses the protein and constitutes the core region, with the other regions well exposed to the solvent packing around the core. This core region might be involved in the process that occur during Tat internalization and certainly requires a structural change for this hydrophobic environment. The basic region (region IV) adopts an extended structure while regions II, III, V and VI have *β*-turns except for Tat Mal, which has an *α*-helix in region V. It is interesting to note that the NMR spectra of Tat variants show a low chemical shift dispersion indicative of a rather flexible structure, which might be a prerequisite for its ability to cross membranes.

In conclusion, structural studies carried out on Tat variants with biological activity show that Tat variants have a similar folding in aqueous solution characterized by a core region composed of a part of region I, which is surrounded by the other regions that are well exposed to solvent. Mutations observed between Tat variants from different HIV-1 subtypes induce local structural variations such as the presence in region V of an *α*-helix in Tat Mal instead of two *β*-turns in Tat Bru and Tat Eli. Tat is rather flexible, and its folding can dramatically change between aqueous and hydrophobic environments.

### Extra-cellular functions of Tat

In addition to the major role of transcriptional activation of viral gene expression, Tat has been implicated in a number of extra-cellular functions during HIV-1 infection. Several studies have suggested that Tat plays a role in viral infectivity and contributes to HIV-1 pathogenesis [20]. For example, immature dendritic cells exposed to exogenous Tat mature and upregulate key co-stimulatory molecules such as CD40, CD80, CD86, lymphocyte function-associated antigens, major histocompatibility complex (MHC) class I and II, lymphotoxin, chemokine (C-C motif) ligand (CCL) 3, CCL4, CCL5, interleukin (IL)-12 and tumor necrosis factor (TNF) [51].

#### Interaction of Tat with integrins and its role in Kaposi's Sarcoma

The first extra-cellular role postulated for Tat was in its direct contribution to Kaposi's sarcoma (KS) associated with AIDS [27,53]. KS is an unusual neoplasm that is typically an indolent disease caused by the human herpesvirus-8 (HHV-8), affecting the skin of elderly males, and is not life threatening. However, AIDS-related KS (AIDS-KS) is dramatically more frequent and more aggressive [54]. Early experiments with transgenic mice with the *tat *gene showed that they rapidly developed dermal lesions resembling KS [55]. Consistent with this finding, exogenous subtype B Tat was shown to stimulate the growth of cells of mesenchymal origin derived from Kaposi's sarcoma lesions of AIDS patients, and was inhibited by anti-Tat antibodies [27]. B Tat also induces the growth and locomotion of primary endothelial cells activated with inflammatory cytokines, in particular, interferon (IFN)-*γ*, TNF and IL-1*β*, which are increased in the blood and lesions of AIDS-KS individuals. IFN-*γ*, TNF and IL-1*β *also augment the synthesis and release of basic fibroblast growth factor (bFGF) from the spindle cells of KS lesions and induce its production from endothelial cells [56,57]. *In vivo*, bFGF exists primarily bound to heparan sulfate proteoglycans, protected from proteolytic degradation, at the surface of cells and extra-cellular matrix, with only a fraction being found in soluble form. Tat, through its conserved basic region, competes with bFGF for heparin-binding sites, increasing soluble bFGF to concentrations that promote spindle cell and endothelial cell growth [56,57] and upregulates the integrins *α*_5_*β*_1 _and *α*_v_*β*_3_, receptors for fibronectin and vitronectin, respectively, both of which are highly expressed in AIDS-KS [58]. One of the similarities between fibronectin, vitronectin and subtypes B and D Tat is the presence in the C-terminal domain of Tat of an RGD motif, which represents the principal cell attachment moiety recognized by integrin receptors. Engagement of integrins during endothelial cell adhesion regulates their migration, tissue organization, matrix remodeling, and, with receptors for soluble factors, survival, differentiation, and proliferation. Therefore, Tat, by engaging with integrin receptors via its RGD motif, promotes the locomotion of spindle cells and activated endothelial cells and provides the adhesion signal they require in order to grow in response to bFGF [59]. This motif has also been implicated in inducing the migration of monocytes and neutrophils through integrins *α*_5_*β*_1 _and *α*_v_*β*_3 _[60]. Mutations in this RGD motif or antibodies derived against this motif prevent the attachment of Tat to integrins [59]. Interestingly, not all Tat subtypes posses this motif, indicating possible subtype specific responses to HHV-8 in HIV-1-infected individuals (Figure 1).

#### Tat and HIV-1 associated dementia

Tat is also a potent chemoattractant for macrophages and monocytes and dendritic cells, but not lymphocytes [16,61]. Region II of Tat has positions of amino acid similarity with key residues in *β*-chemokines critical for chemokine receptor binding and signal transduction [61], including a CCF/Y motif at positions 30–32, a strongly conserved Ile^39 ^and a SYXR motif at position 46–49. B Tat induces chemotaxis of monocytes, but not lymphocytes through a CCR2-dependent mechanism that is dependent upon the integrity of the ^30^CC motif of Tat [16,61]. The C31S mutation found in C Tat variants abrogates its ability to act as a chemoattractant for monocytes as it fails to bind CCR2 and induces a transient flux in cytosol Ca^2+ ^[61].

The role of Tat in the development of neurocognitive impairment remains controversial [62,63], but there is evidence of Tat mediating neurotoxicity through its regions II and IV [64,65]. Tat has been detected in postmortem HIV-1 encephalitic central nervous system (CNS) tissue in various infected cells [66,67] as well as in uninfected oligodendrocytes [68]. It is interesting to note that in India where the C subtype is prevalent, the HIV-1 associated dementia is rare [69] and this could be due to the C31S mutation [61]. Nevertheless, despite extensive *in vitro *research and * in vivo *animal studies demonstrating a potential role for Tat in HIV-related CNS impairment, no study to date has directly quantified the *in vivo *levels of secreted Tat in the CNS as Tat is rapidly degraded post-mortem [67]. In a mouse model of brain toxicity, after a single intraventricular injection of Tat, macrophage infiltration, progressive glial activation, and neuronal apoptosis were observed over several days, while within 6 hours Tat was undetectable [70]. Tat also crosses the blood-brain barrier (BBB) and enters the CNS where it has toxic consequences [71]. It interacts with microglia, astrocytes and brain endothelial cells, increasing the expression of inducible nitric oxide synthase and release of nitric oxide [72] and TNF [14], as well as disrupting tight-junction distribution, increasing the blood brain barrier (BBB) permeability [73]. Tat also exerts a neurotoxic effect on hippocampal neurons by disinhibiting Ca^2+^-permeable N-methyl-D-aspartate (NMDA) receptors from Zn^2+^-mediated antagonism, thereby potentiating the NMDA-mediated death [74]. Subtype C Tat is less neurotoxic than subtype B Tat as a result of the C31S mutation with experiments underway to explain this effect [13].

The influence of Tat on the transcription of TNF from monocytes and microglial cells is particularly important in HIV-1 pathogenesis [14] with patients suffering from HIV-1-associated dementia (HAD) having increased expression of TNF and TNF receptors on activated macrophages and monocytes in both the white matter of brain tissue and sera [75]. TNF opens a paracellular route for HIV invasion across the BBB [76], induces the expression of adhesion molecules on astrocytes and endothelial cells [77] and induces the release of chemokine factors from monocytes and microglial cells allowing HIV-1 infected monocytes and macrophages to transmigrate into the CNS [75]. However, TNF also has neuroprotective effects, such as upregulating the production of CCL5 from astrocytes and Bcl-2 from neurons [75], illustrating the multifactorial cause of the disease. B Tat upregulates TNF production from microglial cells and monocytes through a calcium dependent mechanism that involves an increase in intracellular Ca^2+ ^through L-type calcium channels [14]. Subtype C Tat, which fails to induce an intracellular calcium flux due to its C31S mutation, is still able to induce TNF production, although at much reduced levels [14]. The key checkpoint in TNF protein production in monocytic cells is the transcriptional activation of the gene where histone acetyltransferases and chromatin remodeling play critical roles in enhanceosome formation and are required for TNF gene activation. Both subtype B and C Tat aid in these functions, but the mutation of F/Y32W present in CRF_AE Tat interferes with chromatin remodeling of the TNF locus and with the recruitment of p300/CBP-associating factor to the TNF promoter, resulting in lower levels of TNF gene expression and protein production in T cells [19]. The effect of CRF_AE Tat on TNF production from monocytes has not yet been evaluated.

#### Apoptosis and the role of Tat

The hallmark of disease progression in HIV-1 infected individuals is an increased virus load [78] and the progressive loss of CD4^+ ^T cells [79]. Apoptosis, autophagy and activation-induced cell death (AICD) are known to be involved in this process [80-82]. Co-culture experiments of HIV-1 infected and uninfected cells have shown that while HIV-1-infected cells are resistant to HIV-induced death, uninfected bystander CD4^+ ^T cells undergo apoptosis [83]. Some studies have suggested that Tat induces AICD and has no effect on resting CD4^+ ^T cells [84,85], whereas others have shown that activation is unnecessary and Tat can directly induce apoptosis in resting CD4^+ ^T cells [14,15,86,87]. However, no study has addressed the role autophagy may play in Tat-induced apoptosis, although two Tat studies used serum deprivation as a means to initiate apoptosis [14,15]. During starvation, autophagy contributes to the maintenance of cellular homeostasis by maintaining an amino acid reserve for glucogenesis and for the synthesis of essential proteins by targeting cell organelles and aggregates of long-lived proteins for degradation and recycling. However, it may also result in autophagy-associated cell death [88]. The proteins LC3B-II, Beclin I and ATG7 are essential for the latter. Beclin-1 possesses a BH3 domain that interacts with the BH3 receptor domain of the anti-apoptotic proteins of the Bcl-2 family. BH3-only proteins can induce autophagy by competitively disrupting the interaction of Beclin-1 with Bcl-2/Bcl-X_L_, linking the apoptosis and autophagy machinery. One such BH3-only protein, Bad, is known to be activated upon the withdrawal of growth factors [88].

Tat also induces apoptosis by binding to tubulin at the pharmacological site of paclitaxel, enhancing tubulin polymerization [18] and preventing depolymerization [89]. Tubulin polymers form microtubules necessary for cellular morphology, intracellular organelle distribution, chromosome migration during mitosis, cell differentiation, as well as intracellular transport and signaling [90]. Inhibition of microtubule dynamics induces M arrest, mitotic spindle assembly checkpoint activation, Bcl-2 phosphorylation, c-and Jun NH(2)-terminal kinase activation, leading to apoptosis. Furthermore, as microtubules serve as scaffolds for signaling molecules that regulate apoptosis, such as Bim, disruption of microtubule dynamics releases these signaling molecules from microtubules, which then induce mitochondrial membrane permeabilization resulting in the release of critical pro-apoptotic intermembrane space effectors into the cytosol such as cytochrome *c*, apoptosis-inducing factor, Smac/Diablo, Endo G, and pro-caspases [91]. Regions II and III of Tat including the conserved Cys^37 ^and Phe^38 ^are crucial to Tat-tubulin interactions [89]. This region differs from those present in the tubulin-binding domains of conventional microtubule-associated proteins, which typically contain positively charged residues [92]. It is possible that the basic region III of Tat provides the positive charge necessary to neutralize the negatively charged C-termini of tubulin promoting microtubule assembly. The glutamine-rich region V may also play a role in providing the structural conformation required for the Tat-tubulin interaction [14]. In a study of two subtype D 86-residue Tat proteins, it was found that mutations in this region that disturb the formation of an *α*-helix reduced the ability of Tat to bind and polymerize tubulin [14]. Further evidence for this interaction was provided in a comparison of long versus short Tat in inducing CD4^+ ^T cell apoptosis [15]. The short form was less effective than the long form [15]. In the NMR study of the full-length 99 residue Tat Eli, the C-terminus of Tat masks the *α*-helix of the glutamine-rich region [38], possibly reducing this Tat's ability to bind to tubulin.

Tat is also capable of inducing apoptosis in Bim^-/- ^cells [89]. Another pathway by which Tat has been shown to induce the apoptosis of bystander CD4^+ ^T cells is by upregulating Fas ligand (CD178) expression in both infected and uninfected bystander cells [14,15,93]. HIV-1-infected individuals have CD4^+ ^and CD8^+ ^T cells that are more susceptible to CD178-induced apoptosis. Furthermore, CD4^+ ^T cells from HIV-1-infected individuals overexpress Fas (CD95), and the proportion of these increases with disease progression [94]. Therefore, the upregulation of CD178 by Tat may lead to increased apoptosis in the antigen-responding T cells that are overexpressing CD95 [94]. In the only comparison of long versus short Tat proteins ability to induce CD4^+ ^T cell apoptosis, it was shown that the short 86-residue form of Tat upregulates more CD178 mRNA leading to an increases in caspase-8 that was not observed with the full length form [15], highlighting the importance of the C-terminus of Tat.

### Development of an HIV-1 vaccine using Tat

This review will focus on vaccine approaches using full Tat. The difficulty in reviewing all the vaccine approaches that have included Tat or parts of Tat with other HIV proteins is to determine if the effect observed is related to Tat. A good pharmaceutical practice should be to test each active principle separately before testing together to see if a synergic effect is possible. Furthermore it is important to note that stability of a vaccine in solution for at least one month is mandatory for a vaccination campaign. Adherence to these criteria would reduce significantly the number of vaccine projects actually developed against AIDS and would allow one to focus on vaccines that have a chance to be efficient in the field.

Biologically active Tat appears to be a safe approach as indicated by safety studies carried out on monkeys in which no local or systemic toxicity or adverse effects were observed [95-99]. The two main vaccine strategies against Tat up to now use a short, 86 residue version of a B-subtype European Tat variant that is either inactivated [95] or has full activity [96]. These two approaches were tested on macaques followed by a homologous SHIV-1 challenge [96,100]. A significant decrease of viremia was observed in these two studies carried out respectively on Cynomolgus [96] and Rhesus macaques [100], without showing complete protection during primary infection. Another study showed a long term control of infection following SHIV-1 challenge on Tat vaccinated Cynomolgus macaques [101].

#### Conflicting results regarding Tat vaccination

It is interesting to note that conflicting results appears in Tat vaccine studies on macaques since no protection was observed with a SIV challenge [102] or a vaccination with a recombinant virus coding for a Tat-Rev protein [103]. These conflicting results could be explained by different immunization regimens, viral stocks, routes of viral challenge, and animal species. The difference between SIV Tat and HIV-1 Tat in the first study and the probability that a Tat-Rev recombinant protein does not have the native Tat folding or the native Rev folding for the second study may explain the absence of protection. More puzzling, however, are the results of two other studies using similar viral vectors expressing Tat, Env and Gag that gave opposite conclusions. One study showed the efficacy of vectored Tat, but not Gag and Env [104], while another study showed efficacy of vectored Gag and Env, but not Tat [105]. The main difference in the two studies was that one used a homologous challenge with the Tat Bru sequence in both the vaccine and in the SHIV [104] while the other used a heterologous challenge with the Tat Bru sequence in the vaccine and Tat JR in the SHIV [105]. HIV-1 JR and HIV-1 Bru are B subtypes (Figure 1), but their Tat sequences have non-conservative mutations inducing conformational changes [43]. Theses mutations between the vaccine and the virus used for the challenge might explain the lack of efficacy of the Tat vectored vaccine in the second study [105]. The second study resembled more closely reality since a vaccinated person would not likely be exposed to a homologous virus infection. However, it is not clear why the investigators in the same experiment used a homologous Gag and Env [105].

Over the last 20 years, HIV-1 vaccine studies that target the HIV-1 envelope proteins have been tested using a homologous SHIV/macaque model and have met with some success [106]. However, this was not followed by success in clinical trials [107]. This is likely due to the high genetic diversity of HIV-1, and this is a reason why heterologous SHIV challenge in macaques, with a genetically distinct virus, should be used to determine if a vaccine can be effective against HIV-1 infection in humans [106]. If a successful homologous SHIV challenge is used to provide support for Tat vaccination *in vivo*, then the development of a worldwide Tat vaccine in humans need to additionally take into account the genetic diversity of HIV-1 Tat proteins. In this regard, it is important to note that immunization with the B subtype Tat Bru does not stimulate an efficient response against Tat variants from A and C subtypes [41].

#### Tat antibodies in human sera

The interest in developing a Tat vaccine rose with the discovery that seropositive long-term non-Progressor (LTNP) patients had a higher level of Tat antibodies than seropositive Rapid Progressor (RP) patients [49,50,95,108,110,111]. It is notable that with a sera dilution of 1:1000, Tat Bru is recognized by only 30% of the RP patients in Europe [95] and only 10 to 14% of RP patients in Africa [111]. This percentage can reach up to 50% in Africa if other Tat variants from subtypes A, C and D are tested [17]. This result outlines again how mutations in Tat variants can affect immunogenicity, but it shows also that a large amount of seropositive patients are unable to recognize Tat. Furthermore Tat antibodies in African RP patients have no effect on their progression to AIDS [34]. Thus for a majority of HIV-1 infected patients, Tat is not recognized and although this protein is present in the circulating of infected individuals, those who recognize Tat can apparently not neutralize this protein.

#### Low cross recognition between Tat variants

Only region IV is well conserved among Tat variants (Figure 1), but this region is not recognized by sera from HIV-1 infected patients [17]. Why the basic region of Tat is not recognized by the human immune system could be due to sequence similarity of the basic region of Tat (^48^GRKKRRQRRR) with epitopes found in human proteins such as protamine (^24^RSCRRRKRRSCR). It is interesting to note that two thirds of new born children from HIV-1 infected mother succeed to escape HIV-1 infection that can occur during the delivery or the breastfeeding and generally sero-revert when they are eighteen months old [112]. This high proportion excludes genetic factors that could be due to an innate immunity against HIV. It could be possible that a repression of the immune system to recognize Tat may exist in adults, but not among new born children since the full expression of protamine arrives with sexual maturation.

In the other Tat regions that appear to be recognized by the immune system, a high level of mutations exists since 40% of Tat can be mutated without loss of activity [17]. It is clear that the discrepancy in two studies on the same cohort regarding the number of patients who recognize Tat in Uganda [17,111] is related to the absence of cross recognition by antibodies to African Tat variants when they used to detect an European Tat variant [111]. This finding was previously reported with vaccination of rabbits with different Tat variants [41], and it illustrated that a Tat vaccine using a European variant would be inefficient in Africa where the majority of the HIV infected individuals are located.

#### Innate and acquired immunity

More attention should be placed on the natural immunity against HIV. Natural immunity against HIV-1 is observed in a low proportion of the human population and encompasses different mechanisms ranging from chemokine mutations to the capacity to produce neutralizing antibodies against the HIV-1 envelope [112,113]. Natural immunity can be innate or acquired, the latter being of course the most interesting for vaccine development. Patients with natural immunity against HIV-1 can be exposed and still remain persistently seronegative (EPS), or they can be seropositive and remain long term non progressors (LTNP). In most cases, this natural immunity turns out to reflect innate immunity. However, there is a very rare category of EPS patients highly exposed to the virus who are resistant to HIV-1 due apparently to an acquired immunity. This was revealed by EPS patients in Kenya who were sex workers and who became seropositive and then developed AIDS after a lapse in sex work, showing that their former resistance to HIV-1 was not innate [114]. Kenyan sex workers who are EPS had been intensely studied, and their resistance to HIV-1 appears to be related to their capacity to develop an efficient CD8 T cell response against HIV-1 [115]. However, the paradox is that the CD8 T cell response in EPS Kenyan sex workers is five times lower in magnitude than that of seropositive Kenyan sex workers who ultimately develop AIDS [116]. To make things even more puzzling, studies of similar cohorts of EPS individuals in Ivory Cost, Vietnam and Cambodia show that they have no HIV-1 specific CD8 T cell response but do have natural killer (NK) cell responses [117,118], antibodies against HIV-1 envelop proteins [119], or cellular factors that affect steps of viral entry [120].

#### Acquired immunity against HIV-1 in a cohort in Gabon

During the eighties in Africa, it was observed in a remote area of Gabon called "Haut Ogooué" that seropositive individuals were not developing AIDS and that they ultimately could sero-revert [121,122]. An epidemiological survey was designed and carried out on 750 pregnant women for two years, and 25 were identified as seropositive [122]. From these 25 seropositive women, 23 sero-reverted and became EPS during the two years of the survey. Although EPS patients have normally no detectable virus, it was possible to isolate and clone a HIV-1 strain from one patient called Oyi when she was seropositive [122]. Contrary to other EPS cohort of sex workers or drug users that were constituted many years after the first exposure to HIV, the Gabon cohort was constituted during the primary infection, and this may explain why it was possible to clone a virus. All women infected with HIV-1 Oyi sero-reverted but maintained a CTL response against HIV-1 and had antibodies against P24 [122]. Some women infected by HIV-1 Oyi were also infected by a highly virulent strain similar to HIV-1 Eli [122]. The high proportion of EPS phenotype in this cohort (92%) indicated that the resistance to HIV-1 was probably due to an acquired immunity and not an innate immunity that is statistically observed in less than 5% of the population. Ten years after the publication of the above study, the 23 women remained in good health and traces of HIV-1 infection were no longer detectable in their blood (Eric Delaporte, personal communication). It is interesting to note that HIV-1 infection appears to be very low in Gabon compared to other central African countries [123].

HIV-1 Oyi has genes similar to regular HIV-1 strains except the *tat *gene, which has mutations never found in other Tat variants [43]. Immunization with Tat Oyi raises antibodies in rabbits that were able to recognize different Tat variants even with mis-matched amino acids of up to 38%; this phenomenon has not been seen from immunization with other Tat variants [41]. Tat Oyi appears to induce a humoral immune response against a three-dimensional epitope that is conserved in other Tat variants, and this humoral response could make it possible to neutralize extracellular Tat. Recently, it was shown that Tat Oyi immunization of macaques induced a predominant Th2 immune response while a predominant Th1 immune response was commonly observed after immunization with a non-Oyi Tat [124].

The role of extracellular Tat was not known during the nineteen eighties, and the presence of antibodies against Tat was not tested in this Gabon cohort [122]. However, we recently were able to detect Tat antibodies in a cohort of EPS patients in Vietnam (data not published). Two third of the patients had Tat antibodies characterized by the capacity to recognize Tat variants from the five main HIV-1 subtypes (data not published), while RP seropositive patients recognized mainly Tat variants from one or two HIV-1 subtypes [17].

#### Heterologous SHIV challenge after vaccination with Tat Oyi

Seven rhesus macaques were immunized with synthetic Tat Oyi complemented with an adjuvant, and then a heterologous challenge with the European SHIV BX08 was carried out on Tat Oyi vaccinated macaques and control macaques. Tat Oyi vaccinated macaques had lower viremia compared with control macaques. The most interesting finding was that SHIV infected cells were no longer detectable at 8 weeks post-challenge in Tat Oyi vaccinated macaques. Surprisingly, the macaque that had the lowest viremia had no antibodies against SHIV envelop proteins. This macaque was challenged again, and the animal experienced a short period of seropositivity and sero-reverted [47]. It was, therefore, possible to reproduce experimentally *in vivo *what is observed in the field with EPS patients. This experiment of heterologous SHIV challenge after Tat Oyi vaccination shows that it could be possible to dramatically reduce the level of HIV infected cells in HIV infected patients. Of note, this goal has never been achieved with antiviral treatments.

As a conclusion, a vaccine approach using Tat should take in account the mutations that can occur in Tat variants. Conformational epitopes are essential to obtain cross recognition of Tat variants and therefore a full Tat protein with the second exon to have the right folding. The second exon of Tat elicits immunity against Tat [125], and a long form of the second exon improves cross recognition of Tat variants [52]. However, up to now, only the immunization with a sequence related to the Tat Oyi variant makes possible the cross recognition of Tat variants from the main HIV-1 subtypes, which appears to be one of the characteristics observed with antibodies able to neutralize Tat extra cellular functions.

## Competing interests

The authors declare that their Tat vaccine technology is under licensing agreement with commercial for profit firms.

## Authors' contributions

GRC and EPL were equally involved in drafting and revising the manuscript. Both authors read and approved the final manuscript.
